# Interdisciplinary, Comprehensive Oral and Ocular Evaluation of Patients with Primary Sjögren’s Syndrome

**DOI:** 10.1038/s41598-017-10809-w

**Published:** 2017-09-07

**Authors:** Behzod Tashbayev, Shermin Rusthen, Alix Young, Bente Brokstad Herlofson, Lene Hystad Hove, Preet Bano Singh, Morten Rykke, Lara Adnan Aqrawi, Xiangjun Chen, Øygunn Aass Utheim, Tor Paaske Utheim, Øyvind Palm, Janicke Liaaen Jensen

**Affiliations:** 1 Department of Oral Surgery and Oral Medicine, Faculty of Dentistry, University of Oslo, Oslo, Norway; 2 The Norwegian Dry Eye Clinic, Oslo, Norway; 3 Department of Oral Biology, Faculty of Dentistry, University of Oslo, Oslo, Norway; 4 Department of Medical Biochemistry, Oslo University Hospital, Oslo, Norway; 5 Department of Rheumatology, Oslo University Hospital, Oslo, Norway

## Abstract

A comprehensive evaluation of oral and ocular symptoms and findings in primary Sjögren’s syndrome (pSS) patients may provide valuable information for management. Medical history was obtained from female pSS patients, and sex- and age-matched non-SS patients with sicca symptoms (non-SS sicca controls) as well as healthy subjects without sicca complaints (healthy controls). Oral (Summated Xerostomia Inventory, SXI) and ocular (McMonnies Dry Eye questionnaire, MDEIS, and Ocular Surface Disease Index, OSDI) subjective complaints were recorded. Objective findings including clinical oral dryness scores (CODS), unstimulated and stimulated saliva secretion rates (UWS/SWS), Schirmer I test, tear osmolarity, tear film break-up time (TFBUT), and ocular surface staining (OSS) were determined. The pSS and non-SS sicca controls were extensively troubled by subjective dryness, while the pSS group had higher CODS, significantly lower saliva and tear secretion, shorter TFBUT and higher OSS than both control groups. Furthermore, candida counts were significantly higher in the pSS patients. In the pSS group, subjective oral dryness significantly correlated with ocular dryness (MDEIS: r = 0.5, OSDI: r = 0.413) and SWS was significantly correlated with Schirmer I (r = 0.419). The findings imply that interdisciplinary subjective and objective evaluation of patients with xerostomia and xerophthalmia not only have implications for patient care, but also may guide clinicians in differentiating between pSS and non-SS sicca patients.

## Introduction

Sjögren’s syndrome (SS) is a chronic autoimmune connective tissue disorder characterized by lymphocytic infiltration of exocrine glands, primarily the salivary and lacrimal glands^1^. Exocrine glands in the nose, skin and vagina, as well as in the respiratory and gastrointestinal tracts may also be affected^2, 3^. As for other connective tissue disorders, patients with SS are usually investigated by rheumatologists, however, interdisciplinary management involving oral medicine and ophthalmology is also required^4^. SS is considered primary (pSS) when it develops independently, or secondary (sSS) when another connective tissue disorder has been diagnosed prior to sicca symptoms^5^.

Although the aetiology of pSS is still unknown, environmental and genetic factors have been suggested to play a role^6^. The proposed pathogenic mechanism is an autoimmune reaction that results in a focal infiltration of mononuclear cells in salivary and lacrimal glands^7^. A long-lasting inflammatory process leads to the loss of glandular cells, resulting in reduction, or even complete loss of saliva and tear secretions^8^. The prevalence of pSS has been reported to range from 0.03% to 2.7%^9^ and mainly middle-aged women are affected. More than 95% of the patients present with symptoms of both dry mouth and dry eye, referred to as the sicca complex.

Xerostomia is the subjective sensation of dryness in the oral cavity. Symptoms of dry mouth often include a sticky, dry feeling in the mouth and throat, frequent feeling of thirst, and ulcers may occur in the oral cavity^10^. Patients with dry mouth may have difficulties with articulation, and problems with tasting, chewing and swallowing^11^. As a result of reduced salivary secretion, these patients have a higher risk of developing caries, candidiasis, and mucositis and they may also suffer from bad breath (halitosis) and difficulties wearing dentures^12^. Consequently, dry mouth often results in reduced quality of life^13^.

Dry eye disease (DED), defined by the 2007 International Dry Eye Workshop (DEWS), is “a multifactorial disease of the tears and ocular surface that results in symptoms of discomfort, visual disturbance, and tear film instability with potential damage to the ocular surface”^14^. Symptoms of DED include ocular burning and foreign body sensation, soreness, stinging, irritation, reduced visual acuity, photophobia, double vision, and ocular pain. Burden of DED can vary from mild discomfort in daily activities to incapacitation in physical functioning^14^.

Oral and ocular symptoms in pSS can be relieved with saliva and tear substitutes and stimulants, while involvement of the extra-glandular organs such as kidneys, lungs, skin, joints and muscles may require systemic treatment^15^. The range of symptoms associated with multiple organ involvement make pSS a complex disease to handle, for patients, dentists, rheumatologists and other health workers^16^. Patients with SS have a 6-fold increased risk of developing non-Hodgkin’s lymphoma (NHL), and the lifetime risk of developing lymphoma is 5–10%^5^. Interestingly, findings from salivary gland biopsies such as the presence of germinal centres may be a predictor for lymphoma development^17^. Interdisciplinary, comprehensive evaluation of pSS including rheumatological examinations, and detailed examinations of dry mouth and dry eyes together with histopathological investigations may therefore have an important role in subgrouping the patients, and in turn benefit the choice of treatment strategies. Furthermore, detailed knowledge about the interplay between symptoms and findings of oral and ocular dryness is lacking.

The aim of this study was to comprehensively investigate oral and ocular symptoms and findings in pSS patients and to perform comparisons with age- and gender-matched healthy and sicca control groups. A further aim was to explore possible correlations between oral and ocular findings in the pSS group.

## Methods

### Study participants

This cross-sectional study involved collaboration between the Department of Rheumatology, Oslo University Hospital (OUH), the Dry Mouth Clinic, Faculty of Dentistry, University of Oslo, and the Norwegian Dry Eye Clinic, and was conducted in the period from August 2015 to June 2016. All the pSS patient participants were between 30–80 years of age and fulfilled the classification criteria of pSS according to the American-European Consensus Group (AECG)^18^. In order to obtain a homogenous patient group, all patients were required to have anti-SSA antibodies in serum, whereas a positive salivary gland biopsy was not required for inclusion in this study. In total, 34 female pSS patients were included in the study. A control group of 32 healthy, age- and gender-matched subjects served as a healthy control group. The exclusion criteria for the healthy controls were as follows: a feeling of dryness in the mouth or eyes, presence of systemic disorders with oral or ocular involvement, and a history of surgical procedures that might affect secretion from the glands. In addition, a control group consisting of 17 non-SS patients with sicca symptoms was included. The patients in this group all suffered from dry eyes and dry mouth and were anti-SSA/SSB negative. They had previously been referred to the last author for labial salivary gland biopsy, and all had a focus score < 1. Thus, the non-SS sicca control group consisted of patients with dry mouth and dry eye complaints and findings who were thoroughly evaluated for pSS, but who did not fulfill the classification criteria as they were not positive for autoantibodies and had a negative salivary gland biopsy.

The study protocol was approved by the Norwegian Regional Committee for Medical and Health Research Ethics (REK 2015/363). The study was performed in compliance with the tenets of the Declaration of Helsinki. Prior to participation in the study, written informed consent was obtained from all participants. The data was de-identified prior to analysis.

### Clinical evaluation

#### Dry mouth examination

All study participants were instructed to refrain from eating, drinking and smoking one hour prior to their appointment at the Dry Mouth Clinic. Patient histories were recorded electronically. All other clinical findings were recorded in questionnaires within the University Health Network database, and data were consolidated and exported for statistical analysis as described by Oeyri and co-workers^19^. The participants were evaluated for subjective and clinical manifestations of oral dryness. All participants answered six questions defining symptoms of oral and ocular dryness from the AECG criteria for pSS^18^ and all pSS patients were asked the standard xerostomia question “How often does your mouth feel dry?”^20^. Participants were also asked to respond to the five statements that make up the Summated Xerostomia Inventory – Dutch Version (SXI-D)^20^. SXI-D is a shortened version of the Xerostomia Inventory (XI)^21^ questionnaire used to determine the severity of xerostomia. The SXI-D sum score can range from 5 to 15, a maximum sum score being indicative of participants experiencing very severe problems related to dry mouth.

The participants underwent a thorough oral clinical examination. The Clinical Oral Dryness Score index (CODS) was used to acquire an objective score for oral dryness^22^. The CODS index is determined from 10 different features of oral dryness, and each positive feature scores 1 point for a total ranging from 0–10. Higher scores indicate greater clinical severity of oral dryness. An evaluation of oral dryness was also performed with the sliding mirror test (0 = no friction, 1 = friction and 2 = severe friction). The presence of oral candida was tested by rubbing a sterile cotton swab over two oral mucosal sites: the left cheek and the (anterior part of the) tongue. Samples were inoculated on Sabouraud’s dextrose agar plates, incubated for four days at 37 °C, and growth scored semi-quantitatively; score 0: no growth, score 1: 1–9 colonies (minimal growth), score 2: 10–29 colonies (moderate growth), and score 3: > 30 colonies (severe growth)^23^.

Standardized sialometry was performed on all participants. Unstimulated (UWS) and chewing-stimulated whole saliva (SWS) were collected to determine saliva secretion rates. UWS was collected for 15 min in pre-weighed plastic cups chilled on ice. Participants then chewed a paraffin wax tablet (Paraffin pellets, Ivoclar Vivadent, Shaen, Lichtenstein) for approximately 30 s, swallowing any saliva in the mouth. Thereafter, SWS was collected for 5 min while the participants continued chewing, expectorating the saliva regularly into a new pre-weighed plastic cup chilled on ice. Saliva samples were weighed and saliva secretion rates calculated for both UWS and SWS (g/ml = ml/min).

#### Dry eye examination

At the Norwegian Dry Eye Clinic each participant answered two dry eye disease specific questionnaires: the McMonnies Dry Eye questionnaire (MDEIS)^24, 25^ and the Ocular Surface Disease Index (OSDI) questionnaire^26^. MDEIS was designed as a screening tool to distinguish patients with dry eye disease from a normal population, and it is based on the absence or presence of dry eye specific symptoms^27^. Scores range from 0 to 45, and any score over 14.5 generally indicates the presence of dry eye disease. OSDI is a 12-item questionnaire designed to provide a rapid assessment of the symptoms of ocular irritation consistent with dry eye disease and their impact on vision-related functioning. The OSDI scale ranges from 0 to 100, with higher scores representing greater disability due to eye symptoms. The overall OSDI score defines non-DED (0–12 points), as well as mild (13–22 points), moderate (23–32 points), and severe (33–100 points) DED^28^.

After completing the questionnaires, the subjects underwent a comprehensive ophthalmic examination in the following order: tear osmolarity measurement using TearLab Osmolarity System (TearLab Corp, San Diego, CA)^29^, tear film break-up time (TFBUT) measurement^30, 31^, ocular surface staining recorded according to the Oxford grading scheme^32^, and assessment of tear production using Schirmer I test (i.e without anaesthesia)^30^. The TearLab Osmolarity System has been recognized as a clinical diagnostic tool in dry eye disease with the threshold value of ≥308 mOsm/L indicating dry eyes^33^. TFBUT indicates tear film stability and values ≤ 10 mm/sec defines instable tear film causing ocular dryness. Schirmer I test is routinely used to assess ocular surface dryness. Wetting of only 0 to < 10 mm of the Schirmer strip after 5 min is generally regarded as abnormal, suggesting dry eye disease^34^ while 5 mm/5 min is the cut off for pathology regarding the pSS classification criteria. Ocular surface staining is used to evaluate ocular surface damage in potential DED. The Oxford grading scheme quantifies the estimated damage on a scale from 0 to 15. A higher score implies more ocular surface damage in exposed interpalpebral cornea and conjunctiva^32^. Both eyes of each subject were examined and the average values from both eyes were used for analyses.

The statistical analyses were performed with commercial software SPSS for Windows, version 22 (IBM, Chicago, IL). Missing values in questionnaires were replaced with the mean value of all valid responses. The normal distribution of variables was verified by the Shapiro-Wilk tests. The mean of every oral and ocular measurement for the three groups was compared. One-way ANOVA was used in the intergroup comparison of parameters with normal distribution, while Kruskal-Wallis H test was used in parameters without normal distribution. Correlations between variables were undertaken by using Pearson correlation coefficient or Spearman’s rank correlation analyses. A p-value of < 0.05 was considered to be statistically significant throughout the study. The results of the analyses are presented as mean ± standard deviation (SD).

## Results

Comparison of age, height, weight, and ethnicity did not show any statistically significant differences between the pSS patient group and the two control groups (Table 1). The vast majority of participants in all groups were ethnic Scandinavians and the marital status of the participants did not differ between the groups. In all three groups; pSS, non-SS sicca controls and healthy controls, some subjects used drugs that may possibly influence saliva and tear secretion; antidepressants (2, 2, 1), anti-allergics (5, 8, 6), antihypertensives (6, 3, 1), analgetics (11, 8, 0), and hypnotics (2, 3, 1), respectively. Smoking prevalence among pSS patients, non-SS sicca controls and healthy controls was 12%, 24%, and 6%, respectively.

**Table 1:** Characteristics of the pSS, non-SS sicca controls and healthy controls. The results of the analyses are presented as mean ± standard deviation (SD) and n (%).

Participant characteristics	pSS group (n = 34)	Non-SS sicca control group (n = 17)	Healthy control group (n = 32)	P value
Age (y)	52.9 ± 11.9	52.7 ± 11.3	49 ± 11.5	0.348
Range	32–72	34–76	32–79	
Height (cm)	169 ± 6	167 ± 6	168 ± 5	0.494
Range	153–180	158–178	157–179	
Weight (kg)	72.7 ± 15.2	73.6 ± 15.8	66 ± 10.6	0.086
Range	51–120	60–120	50–90	
Ethnicity				0.653
Caucasian	33 (97%)	15 (88%)	31 (97%)	
Other	1 (3%)	2 (12%)	1 (3%)	
Education				0.053
Basic education	3 (8.8%)	0 (0%)	1 (3.1%)	
Secondary education	14 (41.2%)	7 (41.2%)	5 (15.6%)	
Higher education	17 (50%)	10 (58.8%)	25 (78.1%)	
Smoking status				0.201
Current smoker	4 (12%)	4 (24%)	2 (6%)	
Current non-smoker	30 (88%)	13 (76%)	30 (94%)	
Marital status				0.206
Married/cohabiting	20 (61%)	14 (82%)	20 (62%)	
Unmarried	7 (21%)	2 (12%)	6 (18%)	
Divorced/widow	6 (18%)	1 (6%)	6 (20%)	
Occupation				0.313
Working full/part-time	19 (55.9%)	9 (52.9%)	28 (87.5%)	
Unemployed	1 (2.9%)	1 (5.9%)	1 (3.1%)	
Sick leave/rehabilitation	11 (32.4%)	5 (29.4%)	0 (0%)	
Student	0 (0%)	0 (0%)	1 (3.1%)	
Retired	3 (8.8%)	2 (11.8%)	2 (6.3%)	

### Oral findings

Subjective oral complaints were pronounced in the pSS and non-SS sicca control groups; they responded positively to an average of more than four Sjögren’s specific questions, while in the healthy control group, only three persons answered positively to one question each. These questions cover oral as well as ocular dryness. The SXI-D questionnaire focusing on oral dryness, where the minimum score of 5 indicates no oral dryness, demonstrated significantly more severe oral dryness in the pSS and the non-SS sicca control groups as compared to the healthy control group (mean scores: 12.1 ± 2.5, 12.4 ± 1.8 and 5.94 ± 1.0). The SXI sub-questions^20^ yielding the highest number of positive responses by the two dry mouth patient groups were; having a dry mouth often (pSS 75%, non-SS sicca 94%), and often having difficulty eating dry food (pSS 62%, non-SS sicca 47%). Additionally, 59% of pSS and 47% of the non-SS sicca controls, respectively, responded “always” to the standard xerostomia question: “How often does your mouth feel dry”?

**Figure 1:**
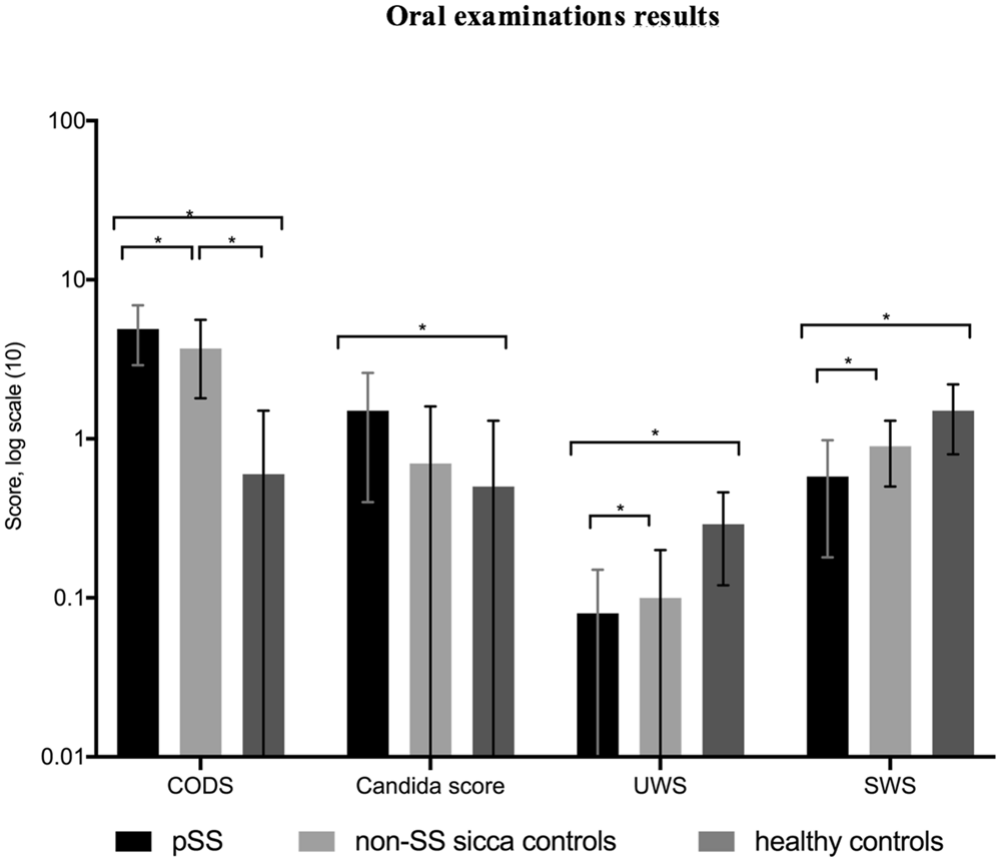
Oral examination results for the pSS, non-SS sicca and healthy control groups are shown in the log scale. CODS – Clinical Oral Dryness Score; UWS – unstimulated whole saliva secretion rate (ml/min); SWS – stimulated whole saliva secretion rate (ml/min). *Level of significance between the groups: p < 0.05 in all parameters. Exact values of variables given in the following order: pSS, non-SS sicca controls, healthy controls. CODS (4.9; 3.7; 0.6), Candida score (1.5; 0.7; 0.5), UWS (0.08; 0.10; 0.29), SWS (0.58; 0.90; 1.50).

Objective clinical results confirmed the subjective findings. According to the CODS index^22^, a significantly higher mean oral dryness score was shown in the pSS group than in the non-SS sicca control group, than in the healthy control group (Fig. 1). In the pSS group, 62% had a CODS value of ≥ 5 vs 35% in the non-SS sicca control group and 0% in the healthy control group. Some of the index items yielded high scores in both the pSS and non-SS sicca groups; Q1) mirror sticks to buccal mucosa (62% of pSS patients vs 65% of non-SS sicca controls), Q2) mirror sticks to tongue (62% of pSS patients, vs 76% of non-SS sicca controls). Other index items differed between the pSS and the non-SS sicca groups. Candida counts were three times higher among the pSS patients compared to the healthy control group. In the pSS patient group, UWS was 25%, and SWS 30% of those in the healthy control group. For non-SS sicca controls, UWS was also 25% compared to healthy controls, while SWS was 60% of healthy control values, whereas and candida scores were similar to those of healthy controls.

In pSS patients, Spearman’s rank analysis revealed positive moderate correlations between SXI and i) the number of positive answers to Sjögren’s specific questions, ii) the standard xerostomia questions, iii) results of the sliding mirror test and iv), and glossy appearance of the palate (Table 2). Furthermore, oral candida scores correlated positively (week to moderate correlations) with the objective oral dryness parameters (Table 3): i) sliding mirror test; ii) mirror sticking to the tongue; iii) lack of saliva pool; and iv) total CODS score. A negative moderate correlation was found between the candida score and UWS and SWS, respectively. For all pSS patients with the highest candida score, the mirror stuck to the tongue, they had a CODS of ≥ 5, a UWS ≤ 0.73 ml/15 min (0.05 ml/min) and all but two had a SWS ≤ 0.66 ml/5 min (0.13 ml/min).

**Table 2:** Table showing significant positive correlations between results of the Shortened Xerostomia Inventory (SXI) and Sjögren’s specific questions, standard xerostomia questions, sliding mirror test and glossy appearance of the palate in the pSS group.

Clinical parameters of oral dryness	Level of correlation (r)	Level of significance (p)
Sjögren’s specific questions	0.49	p < 0.05
Standard xerostomia questions	0.59	p < 0.05
Sliding mirror test	0.41	p < 0.05
Glossy appearance of the palate	0.51	p < 0.05

**Table 3:** Table demonstrating significant correlations between candida score and other objective clinical parameters of oral dryness in the pSS group. UWS–unstimulated whole saliva secretion rate (ml/min); Total CODS– total Clinical Oral Dryness Score; SWS–stimulated whole saliva secretion rate (ml/min).

Objective clinical parameters of oral dryness	Level of correlation (r)	Level of significance (p)
Sliding mirror test	0.39	p < 0.05
Mirror sticking to the tongue	0.38	p < 0.05
Lack of saliva pool	0.36	p < 0.05
Total CODS score	0.43	p < 0.05
Unstimulated whole saliva secretion rate (ml/min)	−0.52	p < 0.05
Stimulated whole saliva secretion rate (ml/min)	−0.53	p < 0.05

### Ocular findings

**Figure 2:**
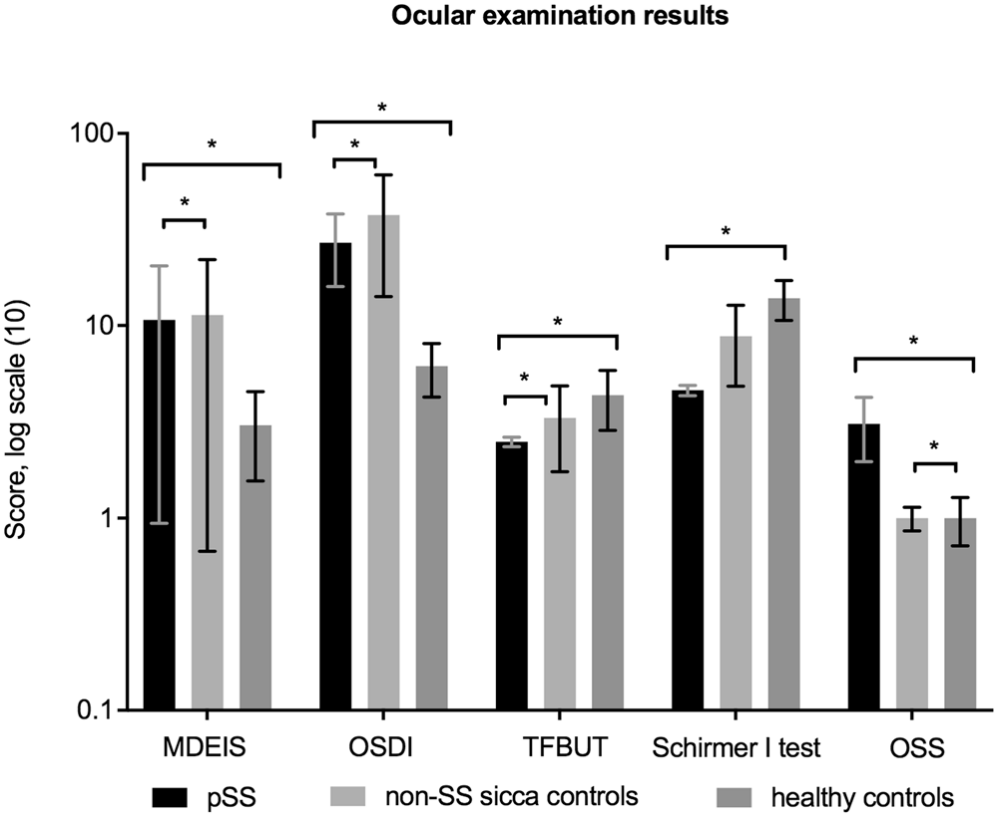
Ocular examination results for the pSS, non-SS sicca and healthy control groups are shown in the log scale. MDEIS – McMonnies Dry Eye Questionnaire, OSDI – Ocular Surface Disease Index, TFBUT – tear film break-up time, Schirmer I test, OSS – ocular surface staining. Intergroup difference is significant at *p < 0.05 level between all groups in all examination results. Exact values of variables given in the following order: pSS; non-SS sicca; HC. MDEIS (17.6; 18.9; 4.1), OSDI (34.8; 54.1; 4.8), TFBUT (2.4; 4.4; 5.4), ST (4.8; 11.6; 16.2), OSS (3.9; 1.1; 0.8).

The results of the ophthalmological examinations are shown in Fig. 2. The pSS patients had more severe subjective dry eye symptoms than the healthy controls as shown by MDEIS and OSDI. A mean MDEIS score of > 14.5 in the pSS group indicates the presence of DED. The results of the OSDI questionnaire showed that patients with pSS had severe DED and subsequently decreased vision-related functionality. Tear osmolarity levels were higher in the patient group compared to the healthy controls (335±22 vs 320±16 mOsmol/L, p = 0.003). Tear film break-up time (TFBUT) in the pSS group was half of that for the healthy control group indicating a less stable tear film, which is also one of the hallmarks of DED. Tear production levels measured with Schirmer I test indicated significant reduction in the pSS group compared with the healthy controls. The pSS group had severe ocular surface staining score, four times more than the healthy controls, demonstrating presence of severe dryness.

Unexpectedly, even if the level of subjective dry eye symptoms was high in the pSS group, it was even higher in the non-SS sicca control group as shown by MDEIS and OSDI. In contrast, objective clinical findings were the highest in the pSS patients. Tear osmolarity levels in the pSS group indicated severe dry eye disease (335 ± 22 mOsmol/L) as compared to the non-SS sicca control group (321 ± 13 mOsmol/L, p = 0.05). There are different studies that have suggested different cut-off values for dry eyes including 316 mOsmol/L for moderate-severe dry eye disease^35–37^. Tear film break-up time (TFBUT) in the pSS group was significantly lower than in the non-SS sicca controls. Interestingly, tear osmolarity levels and staining scores in the pSS group were considerably higher than in the non-SS sicca controls. Actually, tear osmolarity levels and staining scores were similar in the non-SS sicca and healthy control groups.

### Correlations between oral and ocular findings in the pSS group

Analyses revealed the following significant correlations between the results of ocular and of oral examinations: The total SXI score was moderately positively correlated with the subjective evaluation of dry eye measured with both MDEIS (r = 0.53, p = 0.001) and OSDI (r = 0.413, p = 0.015). Schirmer I test values were moderately negatively correlated with candida score (r = −0.43, p = 0.018) and the presence of a lobulated tongue (r = −0.50, p = 0.006), and a moderate positive correlation was seen between Schirmer I test results and SWS (r = 0.419, p = 0.021).

## Discussion

The present study revealed that the pSS patients studied had pronounced symptoms and findings of oral and ocular dryness. In fact, these patients had significantly more clinical features of oral dryness, severely reduced production of stimulated saliva, and increased levels of oral candida when compared with both control groups. The pSS patients also had less stable tear film, reduced tear production levels and more damaged ocular surfaces compared to the non-SS sicca and healthy controls. The non-SS sicca control group had similarly high levels of oral complaints and the same low UWS secretion rates as pSS patients. Unexpectedly, subjective ocular complaints in this group were even higher than in the pSS patients. Importantly, ocular staining in this positive control group was not elevated compared to healthy controls.

### Dry mouth parameters

In our study, 70% of the pSS patients compared to 6% of the healthy controls had an unstimulated salivary secretion rate of 1.5 ml/15 min or below. This is compatible with the classification criteria for pSS^18^. In general, the patients with pSS as compared to the healthy controls suffered largely from subjective dry mouth symptoms as revealed by the Sjögren’s specific questions as well as the SXI-D questionnaire. The subjective findings of oral dryness in the pSS patient group (average SXI score) corresponded well with that reported by Wang *et al*. (SXI of 11 in pSS patients)^38^. However, the dryness scores were much higher than in various populations reported by Thomson *et al*.^20^ in the original paper on the use of SXI, underlining the high degree of dry mouth complaints among pSS patients. On the other hand, the average CODS value in the pSS patient group in the present study was somewhat lower than reported by Osailan *et al*.^22^ in 25 pSS patients. As both studies included a relatively low number of pSS patients, small variations are to be expected. Regarding the presence of candida in pSS patients, the findings in our study are in accordance with Schinozaki *et al*. 2012^39^ who found colonization by candida species to be higher in xerostomic patients than in controls and that *Candida albicans* was the most frequently isolated species. However, an interesting new finding in our study was that in pSS patients, candida scores had positive correlations with many measures of dry mouth such as the sliding mirror test, mirror sticking to tongue, lack of saliva pool, the total CODS, and negative correlations with both unstimulated and stimulated whole saliva secretion rates. Furthermore, SXI revealed positive correlations with the number of positively-answered Sjögren’s questions, the standard xerostomia question, the sliding mirror test and a glossy appearance of the palate. Although each single test showed weak to moderate correlations, the results taken together are of significant clinical importance. Therefore, it may be suggested that a standard set of questions related to oral dryness like the SXI combined with clinical measures such as UWS, SWS, CODS, and candida scores, may successfully identify the pSS patients with the highest oral disease burden.

Subjective and objective findings in the non-SS sicca group did not differ largely from the pSS group. The main difference was the higher mean CODS value in the pSS group. All patients in the non-SS sicca group were referred to the last author due to a suspicion of having pSS. As they all were autoantibody negative and they all turned out to have a negative salivary gland biopsy, they did not receive any specific diagnosis. The lack of a diagnosis may be a bigger stressor to patients with severe sicca symptoms compared to those being diagnosed with pSS, even if there is currently no good therapeutic treatment available. This may partly explain the high number of subjective complaints seen in the non-SS sicca controls.

### Dry eye parameters

As mentioned, dry eye disease in SS is a component of the sicca complex and a characteristic symptom of the disease. In the present study, patients with pSS had a high level of subjective dry eye symptoms as measured by OSDI and MDEIS questionnaires. The OSDI questionnaire is used extensively in dry eye disease research and has been recommended as a useful tool for use in pSS clinical trials^40^, whereby a score between 33 and 100 indicates severe DED^28^. The mean OSDI score for the pSS group indicated severe dry eye disease, and 50% of the patients had severe dry eyes according to the grouping criteria of OSDI. The healthy control group had quite low OSDI scores and only one subject had a score above 33 indicating severe ocular dryness. A recent study by Fostad and co-workers^41^ reported increased OSDI and MDEIS scores in patients with xerostomia compared to non-xerostomia patients diagnosed with non-SS dry eye disease. Their study is supported by the current findings regarding the usefulness of OSDI.

A stable tear film is important for maintaining a healthy ocular surface. Disturbance in stability of the tear film due to hyposecretion of the tear components causes increased local evaporation and dryness making the ocular surface epithelium more susceptible to damage. The disturbance in the tear film stability is evaluated with TFBUT. The present study showed a pathological TFBUT (<5 s) in 94% of pSS patients compared to 50% of the healthy controls. These findings are in agreement with a recent study by Szalai and associates^42^ reporting decreased TFBUT and tear production in patients with pSS.

Decreased lacrimal secretion is a characteristic finding of SS. As shown in this study, a pathologically low Schirmer I test result (<5mm/5 min) was found in the majority of pSS patients, while only three (12%) of the healthy controls had low Schirmer I test results. Patients with pSS had only one quarter of the tear production rate measured with Schirmer I test compared to the healthy controls and this might explain increased signs of ocular surface inflammation quantified with ocular surface staining. Decreased lacrimal secretion and less stable tear film lead to damage and consequent death of conjunctival and corneal epithelial cells. The extent of damage of the ocular surface is assessed by surface staining scores. In our study 97% of the patients with pSS demonstrated ocular surface staining scores at pathological levels. The severity of the ocular surface damage was four times higher in this pSS group compared to the healthy controls as shown in the results of ocular surface staining. A similar severity of ocular surface damage measured with the ocular surface staining score has been reported in previous studies addressing dry eye aspects of SS^43–45^. It is noteworthy that also some subjects in the healthy control group demonstrated some signs of dry eye which can be explained by the relatively high prevalence of DED in the general population^46^.

Unexpectedly, subjective dry eye symptoms were higher in the non-SS sicca control group than in the pSS group, implying that pSS patients may be coping better with their long-lasting chronic conditions. It could also explain that not having an accurate diagnosis may lead to overstatement of subjective dry eye symptoms among the non-SS sicca control group. Tear film stability shown by TFBUT values was considerably lower in the pSS group, which can be explained by low production of tear film. Schirmer values in the non-SS sicca control group were significantly higher and above the normal threshold values (≥10 mm/5 min), demonstrating normal tear production rates. Importantly, ocular staining seems to differentiate between pSS and non-SS sicca patients, meaning that if a dry eye patient demonstrates high levels of ocular staining along with symptoms of oral dryness, this patient should be informed about the possibility of actually having pSS and as such should be examined by the appropriate specialists.

### Combination of oral and ocular parameters

The pSS patients were recruited from a rheumatology department. All patients fulfilled the AECG criteria. In order to obtain a homogenous patient group, inclusion additionally required the presence of anti-SSA antibodies in serum. At inclusion, there was no specific selection regarding clinical aspects of pSS such as fatigue, dryness, pain or extra-glandular manifestations, thus ensuring that the patient cohort in this study was not skewed in any particular direction. The correlations found in this study between subjective oral and ocular dryness scores and between tear and saliva secretion rates in pSS patients may therefore be a universal trait, but this may not be true for individual patients. Nevertheless, our findings underscore the need in any pSS patient to investigate the eyes in a dry mouth patient and the mouth in a dry eye patient. Additionally, some of the healthy controls had some positive symptoms or findings, highlighting that the diagnosis of pSS must be set by summing up the results of various symptoms, clinical findings and laboratory findings, both in accordance to classification criteria and in clinical practice.

We found no striking differences between pSS and non-SS sicca patients in the oral examinations except for increased CODS and candida values. Dentists are commonly seeing dry mouth patients and the usual cause is medications^47^. However, if no medication is to be identified as the cause and the patient responds positively to a dry eye question, the suspicion of SS should be raised. Importantly, in the ocular examination, ocular staining seems to differentiate between pSS and non-SS sicca patients, in accordance with the revised pSS criteria^48^.

The pSS patients in this study had relatively few current dental treatment needs (e.g. acute caries lesions) as measured by low levels of the D component of the DMFT (data not shown). However, needs related to treatment of fungal infection and oral dryness were largely unmet. These problems may be due to the shortage of good treatment options available, lack of focus on other aspects than treating caries caused by dry mouth among dentists, and the general lack of focus on oral health as an integrated part of general health among medical doctors^49^. The same argument holds true regarding the low level of focus on dry eye diagnosis and treatment among general practitioners and eye care professionals. Even though attention to dry eye disease has increased over the last few years, many cases may still go undiagnosed.

The novelty of the current study is the extensive interdisciplinary evaluation of oral dryness and dry eye signs and symptoms in pSS patients as compared to both non-SS sicca and healthy control groups. Furthermore, strict patient selection criteria add strength to the study. Broad comparison of signs and symptoms of oral dryness and dry eyes between the three groups provides unique information in the context of defining possible future disease biomarkers for pSS. Moreover, the study has the advantage of reporting results of extensive correlations between findings of oral dryness and dry eyes in the pSS group. However, the study also had some limitations. Firstly, some pSS patients used medications for Sjögren’s syndrome and artificial tear substitutes for dry eyes that may have influenced the accuracy of results of actual dry eye severity. Secondly, in all three groups, some subjects used medications that may influence tear and saliva secretion, although these drugs were quite evenly distributed in the pSS and non-SS sicca control groups. Thirdly, even though no subjects in the healthy control group had dry eye complaints when recruited, some of them demonstrated a mild form of dry eye disease when they were examined, confirming the high prevalence of dry eye in the general population and need for education about dry eye disease^50, 51^.

In conclusion, a Norwegian cohort of pSS patients demonstrated significantly more symptoms and findings of both dry eyes and dry mouth, compared to age- and gender matched healthy controls. For pSS patients, severity of dry mouth correlated with severity of dry eyes in terms of subjective symptom scores and levels of secretion of both saliva and tears. Furthermore, when comparing the pSS patients to the non-SS sicca patients, it became evident that the latter were as troubled by dryness symptoms as the pSS patients but that their objective oral and ocular findings were somewhat less pronounced. In particular, ocular staining differed between the two patient groups. The findings have important implications for patient care and show that the combination of extensive oral and ocular examinations is a key factor to ensure targeted and personalized treatment for pSS as well as non-SS sicca patients.
